# Improvement Strategies, Cost Effective Production, and Potential Applications of Fungal Glucose Oxidase (GOD): Current Updates

**DOI:** 10.3389/fmicb.2017.01032

**Published:** 2017-06-13

**Authors:** Manish K. Dubey, Andleeb Zehra, Mohd Aamir, Mukesh Meena, Laxmi Ahirwal, Siddhartha Singh, Shruti Shukla, Ram S. Upadhyay, Ruben Bueno-Mari, Vivek K. Bajpai

**Affiliations:** ^1^Laboratory of Mycopathology and Microbial Technology, Centre of Advanced Study in Botany, Institute of Science, Banaras Hindu UniversityVaranasi, India; ^2^Laboratory of Molecular Biology, Department of Botany, Dr. Hari Singh Gour UniversitySagar, India; ^3^Department of Energy and Materials Engineering, Dongguk UniversitySeoul, South Korea; ^4^Research and Development (R+D) Department, Laboratorios LokímicaValencia, Spain; ^5^Department of Applied Microbiology and Biotechnology, Yeungnam UniversityGyeongsan, South Korea

**Keywords:** fungal glucose oxidase, bioelectronic devices, biofuel, biosensor, cost effective production

## Abstract

Fungal glucose oxidase (GOD) is widely employed in the different sectors of food industries for use in baking products, dry egg powder, beverages, and gluconic acid production. GOD also has several other novel applications in chemical, pharmaceutical, textile, and other biotechnological industries. The electrochemical suitability of GOD catalyzed reactions has enabled its successful use in bioelectronic devices, particularly biofuel cells, and biosensors. Other crucial aspects of GOD such as improved feeding efficiency in response to GOD supplemental diet, roles in antimicrobial activities, and enhancing pathogen defense response, thereby providing induced resistance in plants have also been reported. Moreover, the medical science, another emerging branch where GOD was recently reported to induce several apoptosis characteristics as well as cellular senescence by downregulating *Klotho* gene expression. These widespread applications of GOD have led to increased demand for more extensive research to improve its production, characterization, and enhanced stability to enable long term usages. Currently, GOD is mainly produced and purified from *Aspergillus niger* and *Penicillium* species, but the yield is relatively low and the purification process is troublesome. It is practical to build an excellent GOD-producing strain. Therefore, the present review describes innovative methods of enhancing fungal GOD production by using genetic and non-genetic approaches in-depth along with purification techniques. The review also highlights current research progress in the cost effective production of GOD, including key advances, potential applications and limitations. Therefore, there is an extensive need to commercialize these processes by developing and optimizing novel strategies for cost effective GOD production.

## Overview

Glucose oxidase (GOD; β-D-glucose:oxygen 1-oxidoreductase; glucose aerodehydrogenase; E.C. 1.1.3.4.) is a very important oxidoreductase enzyme (flavoprotein). Structurally, GOD is a holoenzyme consisting of two identical 80 kDa subunits at the active site that containing a cofactor flavin adenine dinucleotide (FAD). These subunits act as a redox carrier in catalysis. GOD belongs to the glucose/methanol/choline (GMC) oxidoreductase family, which incorporates numerous other industrially imperative catalysts, particularly in the field of diagnostics, for example, cholesterol oxidase, choline oxidase, methanol oxidase, alcohol oxidase, amino acid oxidase, and pyranose oxidase (Ferri et al., 2011). These members of the GMC oxidoreductase family share a homologous structural backbone, including an adenine-dinucleotide-phosphate binding β*αβ*-fold close to their amino terminus and five other segments of conserved sequences dispersed throughout their primary sequences (Horaguchi et al., 2012).

GOD catalyzes the oxidation of β-D-glucose into D-glucono-δ-lactone at its first hydroxyl group using atomic oxygen (O_2_) as the electron acceptor with the synchronous generation of hydrogen peroxide (H_2_O_2_). Both end products break down spontaneously and catalytically. Specifically, D-glucono-δ-lactone is subsequently hydrolyzed slowly by enzyme lactonase to D-gluconic acid (GA), while the generated H_2_O_2_ is broken down to O_2_ and water (H_2_O) by catalase (CAT). GOD catalyzes the oxidation of glucose, according to a ping-pong mechanism (Leskovac et al., 2005).

The overall reaction is given below:

GOD (FAD) + β−D-Glucose → GOD (FADH_2_) + D-Glucono-δ-lactone
GOD (FADH_2_) + O_2_ → GOD (FAD) + H_2_O_2_
β−D-Glucose + GOD (FAD) + O_2_ → GA + GOD (FADH_2_) + H_2_O_2_

GOD is profoundly particularly for the β-anomer of D-glucose, while the α-anomer does not seem, by all accounts, to be a reasonable substrate (Bankar et al., 2009b). Thus, GOD shows bring down exercises while using 2-deoxy-D-glucose, D-mannose, and D-galactose as substrates. Among enzymes currently known to oxidize glucose; GOD is the best-known because of its high degree of specificity. GOD can be obtained from a large number of different sources, including red algae, citrus fruits, insects, bacteria, plants, animals, and fungi. The regular capacity of GOD in these organic frameworks is to act primarily as an antibacterial and antifungal specialist through the generation of H_2_O_2_. Among these sources, fungi have an eminent status and industrially fungal sources have been preferred since the early 1950s (Fiedurek and Gromada, 1997a).

For decades, fungi have been thoroughly inspected for GOD production as cell factories due to their magnificent capacities to use an assortment of carbon sources and to accumulate a large proportion of natural GOD under stressed conditions (Bankar et al., 2009b; Zehra et al., 2015). Because GOD from fungi has applications in a broad spectrum, it must be stable at the higher temperature and for a longer duration so that it can be used economically. Nevertheless, the mechanisms of GOD accumulation by different fungi are not fully understood, even though many successful attempts have been made to improve and optimize fungal GOD production by using genetic modifications and other approaches. The filamentous fungi *Aspergillus* and *Penicillium* serve as industrial producers of GOD at a large scale (Wong et al., 2008; Bankar et al., 2009b), among which *Aspergillus niger* is the most ordinarily used for the industrial yield of GOD (Pluschkell et al., 1996). Various properties of GOD produced by *A. niger* are listed in Table 1, including methods that have been reported for stabilizing GOD, the use of additives, and engineering through site-directed or random mutagenesis coupled to expression in heterologous hosts (Table 1).

**Table 1:** Structural and functional properties of GOD from *A. niger*.

**Features**	**Properties**
Localization	Intracellular (van Dijken and Veenhuis, 1980; Sabir et al., 2007); extracellular (Mischak et al., 1985; Witteveen et al., 1992); both (Stosz et al., 1998).
3D structure	Homodimeric flavoenzyme with two identical 80 kDa subunits (Wohlfahrt et al., 2004); 10–25 wt% glycosylated; serine, glycine, glutamic acid, aspartic acid, and alanine constitute more than 60% of the total amino acids.
Preferred carbon and nitrogen sources for production	Glucose, sucrose, and molasses (Hatzinikolaou and Macris, 1995); peptone and corn steep liquor (Kona et al., 2001; Canli and Kurbanoglu, 2011).
Other preferred medium components for production	n-dodecane, n-hexadecane, and soybean oil (Li and Chen, 1994); ammonium phosphate, sodium orthovanadate, hematin, choline, and Tween 80 (Gromada and Fiedurek, 1996; Bankar et al., 2009a).
Inducers	Glucose (Hatzinikolaou and Macris, 1995); calcium carbonate (Hatzinikolaou et al., 1996); manganese, cobalt, thioglycolic acid and gluconic acid (Liu et al., 2001; Khurshid et al., 2011); EDTA and some metal ion such as Zn^2+^ and Fe^2+^ (Song et al., 2016).
Inhibitors	Ag^+^, Hg^2+^, Cu^2+^, Mg^2+^, CaCl_2_ ions (Nakamura and Ogura, 1968; Toren and Burger, 1968; Singh and Verma, 2013); accumulation of hydrogen peroxide (Kleppet, 1966; Song et al., 2016); arsenite, p-chloromercuribenzoate, phenylmercuric acetate (Nakamura and Ogura, 1968); hydroxylamine, hydrazine, phenylhydrazine, dimedone, and sodium bisulfate (Khurshid et al., 2011); guanidine hydrochloride, urea, SDS (Song et al., 2016).
Bioreactor	Benchtop (Liu et al., 2003); batch (Sarrafzadeh and Jafari, 2008; Canli and Kurbanoglu, 2011); fed-batch (Gu et al., 2015).
Strain improvement	Gamma irradiation (Zia et al., 2012); UV irradiation (Ramzan and Mehmood, 2009; Rasul et al., 2011); random mutagenesis (Haq et al., 2014); screening (El-Hariri et al., 2015); mutagenesis (Fiedurek et al., 1986, 1990; Markwell et al., 1989; Witteveen et al., 1990; Fiedurek and Gromada, 1997a,b); protoplast fusion (Khattab and Bazraa, 2005); site-directed mutagenesis (Holland et al., 2012; Marín-Navarro et al., 2015); chemically treated (Zia et al., 2010); biochemical mutation (Fiedurek and Szczodrak, 1995).
Recombinant glucose oxidase	*H. polymorpha* (Hodgkins et al., 1993); *S. cerevisiae* (Blazic et al., 2013; Marín-Navarro et al., 2015); *T. reesei* (Mu et al., 2006); *P. pastoris* (Guo et al., 2010; Kovačević et al., 2014; Meng et al., 2014; Gu et al., 2015; Qiu et al., 2016); *P. nalgiovense* (Geisen, 1995); *Mucor circinelloides* (Bredenkamp et al., 2010); *Kluyveromyces marxianus* and *Kluyveromyces lactis* (Rocha et al., 2010); *A. oryzae* (Gregg, 2002); *A. nidulans* (Whittington et al., 1990; Luque et al., 2004); *A. niger* (Whittington et al., 1990; Pluschkell et al., 1996).
Optimization by statistical methods	Response surface methodology (Liu et al., 2003; Farid et al., 2013); L27 Taguchi experimental design (Kriaa and Kammoun, 2016); Plackett-Burman design (Bankar et al., 2009a).
Purification techniques	Ammonium sulfate precipitation, gel filtration, Q-Sepharose and DEAE sepharose, DEAE-cellulose ion exchange and Sephadex G-200 size exclusion chromatography (Zia et al., 2013),
Half-life	Approximately 30 min at 37°C. Immobilized GOD would be more effective for application at 37°C. Polyhydric alcohols, including ethylene glycol, glycerol, erythritol, xylitol, sorbitol and polyethylene glycol have shown stabilizing effects (Ye et al., 1998). The lyophilized GOD preparation remains stable for a minimum of 6 months at −20°C (Kelemen and Lantz, 2008).

*Penicillium* species such as *Penicillium amagasakiense* and *Penicillium variabile* have been appeared to show more invaluable energy for glucose oxidation than *A. niger* (Kusai et al., 1960; Witt et al., 1998). Further, GOD from *Penicillium* species has been revealed to be effective because of its high specificity for glucose, high turnover, high stability, safety and long-term stability (Bodade et al., 2010; Konishi et al., 2013). Recently, Courjean and Mano (2011) described the production of a recombinant glucose oxidase (rGOD) from *P. amagasakiense* displaying a more efficient glucose oxidation than the native GOD from *A. niger*. Their results indicated that rGOD from *P. amagasakiense* is a better candidate for development of efficient bioelectrochemical devices. The success of *Aspergillus* and *Penicillium* for industrial production of biotechnological products is largely due to the metabolic versatility of these strains and their generally recognized as safe (GRAS) status. Therefore, fungal GOD has attracted the attention of many biological experts and industrialists and has been the subject of many investigations in various fields for decades because of its numerous commercial applications.

Further, the most remarkable and novel application of GOD has been found to be in the biosensors and biofuels in recent years because GOD can serve as a useful tool for outlining new and more proficient modern procedures. In addition, the GOD combined with cross-linked enzyme aggregates of HRP (horseradish peroxidase) are used in various sectors of food and pharma industry for catalyzing cascade chemical reactions (Zhang et al., 2016; Nguyen and Yang, 2017). Recently, the GOD modified with hydrophilic polymers via *in-situ* RAFT polymerization methodology showed higher enzyme bioactivity. This efficient technique for the surface modification of enzyme can be applied for the amelioration of other biomolecules, and could envision broad application in varied areas such as biomedicine, food industry, and biotechnology (Xu et al., 2017a). Thus, widespread application and increasing demand of GOD in different industries (chemical, pharmaceutical, food, beverages, clinical chemistry, biotechnology, medical diagnostics, environmental conservation, energy, textile, and other industries) have led to the need to produce these compounds extensively (Bankar et al., 2009b). However, common sense utilization of enzymes is frequently restricted by their quick inactivation at extreme temperature, pH or the presence of surfactants upon exposure to elevated temperatures. In the present review, the demands for GOD in human daily life led us to emphasize and update the current status of GOD requirements in daily life. Highly robust GOD forms are desirable for these applications, which justify the development of strategies for increasing the stability of this redox enzyme.

## Currently reviewed potential fungal sources of glucose oxidase

The most common fungal sources of the enzyme GOD from the genus *Aspergillus* are *A. niger* (Liu et al., 2001), *A. tubingensis* (Kriaa and Kammoun, 2016), *A. flavus* (Bhat et al., 2013), *A. terreus* (Anas et al., 2012), *A. oryzae* (Gunasundari, 2014), *A. carbonarius* (Yang et al., 2014), and *A. nidulans* (Witteveen et al., 1990), while those from *Penicillium* are *P. amagasakiense* (Todde et al., 2014), *P. variabile* (Crognalea et al., 2008), *P. chrysogenum* (Konishi et al., 2013), *P. notatum* (Nandy, 2016), *P. funiculosum* (Esmaiilpour et al., 2014), and *P. adametzii* (Mikhailova et al., 2007). Many other species of *Penicillium* have also been reported to produce GOD, such as *P. pinophilum* (Rando et al., 1997), *P. canescens* (Simpson, 2006), *P. fellutanum* (Manivannan and Kathiresan, 2007), *P. glaucum* (Müller, 1928), and *Penicillium vitale* (Dolgiĭ M. L. et al., 1977; Dolgiĭ N. L. et al., 1977). Other reported fungal species include *Talaromyces flavus* (Kim et al., 1990), *Phanerochaete chrysosporium* (Zhao and Janse, 1996), *Alternaria alternata* (Caridis et al., 1991), *Pleurotus ostreatus* (Shin et al., 1993), *Pycnoporus cinnabarinus* (Levasseur et al., 2014), *Rhizopus stolonifer* (Guimarães et al., 2006), and *Flavodon flavus* (Raghukumar et al., 2004).

## Strategies for improving GOD production

For commercial applications, the production of GOD should be cost-effective and economical. Concerning these facts, researchers are putting their continuous efforts in opening and finding various facts and strategies. The large-scale application of GOD through naturally-occurring wild-type fungal strains has been hampered by low fermentation capacity, complicated purification process, and low efficiency. Further, impairments in the application of GOD are associated with an impurity in resources of GOD, which, include CAT, cellulase, and amylase (Frederick et al., 1990).

It is also important to note that during production, GOD can be inactivated by H_2_O_2_. This occurrence results in its accumulation during the oxidation reaction followed by pH reduction, which may accompany the breakdown of D-glucono-δ-lactone to GA, as well as inhibits enzyme production (Klibanov, 1983). It is by and large acknowledged that the appropriateness of a protein for industrial purposes relies upon its high turnover number, heat-stability, and dependability in different media (Klibanov, 1983). Although the expression of GOD is not high in its native state, engineering technologies have improved our capacity for large-scale GOD production. Studies of the enhancement of production and properties of GOD are still receiving a great deal of attention, presumably because of the current and extensive applications of this enzyme. Therefore, as we named the enzyme “Biological God.” Practical perspectives on the use of GOD include its high catalytic activity and substrate affinity as well as high stability (Bhatti and Saleem, 2009). Therefore, the generation of ameliorated versions of GOD is an important biotechnological objective. As an effective biotechnological prospective, several strategies already available for efficient production of GOD are discussed below.

### Solid-state and submerged fermentation strategy

Production of GOD by fungi is commonly performed by solid-state fermentation (SSF) and submerged fermentation (SmF; Mirón et al., 2002, 2010). SmF has been found to be more effective at producing GOD because it is easier to control the environmental factors involved in this technique when compared to SSF. Recently, Kriaa and Kammoun (2016) assessed the variability of GOD by *A. tubingensis* CTM507 based on titers measured under both SmF and SSF in association with growth and substrate-consumption, suggested that comparatively better results on fungal growth were obtained using SmF, whereas SSF represented significantly higher GOD activity (170 U/mL) as compared to SmF (43.73 U/mL). These findings improve our understanding of the phenomena responsible for the better ability of SSF than SmF to produce functional bio-molecules. The studies also explain an SSF process for the low-cost production of important enzymes with attractive properties for commercial applications. These research findings confirmed that *A. tubingensis* was able to exhibit remarkable GOD activity when culture conditions were optimum as compared to other producer strains. However, these traditional methods for the production of GOD have reached their limit. Accordingly, different strategies to overcome these limitations and enhance their production have been explored, including recombination, immobilization, mutagenesis and screening (Zia et al., 2010).

### Immobilization

Immobilized GOD has been widely investigated in the medical, food and environmental fields. Recently, investigations of GOD immobilization mainly focused on electrode modification for biofuel cells (BFCs) or biosensors (Yang et al., 2016). Enzyme-based biosensors have attracted a great deal of attention. Methods used for their production often involve proper surface immobilization of GOD and effective conversion of chemical information to electronic signals. Biotechnological processes in immobilized growing fungal cells, including those for extracellular enzyme production, appears to produce results more favorable than traditional fermentation methods since immobilization enables the repetitive and continuous use of the microbial cells (Correa et al., 2012). Although the native GOD enzyme has attracted interest for its potential in a variety of processes, this enzyme is unstable because of its complex molecular structure. In this respect, knowledge of the intimate interaction between the enzyme and ligands, in vary conditions, is of great importance. For example, Szefler et al. (2016a,b) studied the nature of polyethyleneimine-GOD interactions by docking and molecular dynamics techniques to provide a detailed information on the type, intensity, and frequency of these interactions. Thus, the advancements in the field of bioinformatics during the last decade have also revolutionized the field of GOD immobilization. GOD immobilization techniques provide several benefits, such as faster reaction rates, enhanced stability, and easy product separation from reaction mixtures, reduced wash-out, increased productivity, and catalytic modulation with reduced operation costs.

Recently, for the production of a bioconjugated complex of GOD, surface immobilization of GOD occurred covalently on modified nano-particles of iron oxide. Further, the stability of the immobilized and free enzyme was examined at different pH and temperature as the enzymatic activity measurement. The stability of the enzyme was shown to be enhanced by immobilization. The experimental results confirmed that the enzyme storage stability was improved upon binding to the modified iron oxide magnetic nanoparticles (MIMNs). Moreover, kinetics measurements suggested that the immobilization process had a small effect on the substrate and product diffusion (Abbasi et al., 2016). Thus, for accelerating the rate of GOD catalyzed reactions, a number of bioconjugates are used as catalytic nano-devices, and employed in several biotechnological processes to achieve different goals (**Table 3**). Nano-size materials are currently receiving a great deal of attention for their use as a supportive contribution to these purposes. These nano-materials have the potential for widespread applications because of their simple, non-chemical separation method (Huang et al., 2003), and high enzyme loading capacity owing to their large surface area in contempt of non-porous nature (Park et al., 2011). Bankar et al. (2011) co-immobilized GOD and CAT on an inorganic (non-porous glass beads) support matrix using activators and a cross-linking agent and evaluated its potential uses. They also optimized the immobilization process parameters using statistical techniques to describe improvements in immobilization yield.

In addition, effective GOD immobilization also provides stability and increasing the amount of enzyme activity, resulting in increased electrical power through several enzyme fuel cells (EFCs; Ramanavicius et al., 2005). A variety of methodologies has been investigated for the development of fuel cells, such as direct transfer of the electron between the enzyme and the electrode without the use of redox solution mediator. On the other hand, various electrode-based modified methods have been used in EFCs for the immobilization of GOD, due to their enzyme-dependent performance and the ability to immobilize on the electrodes (Table 2). Among the methods, covalent bonding-base methods are most studies methods, since these methods enable immobilization of enzymes for a prolonged reaction time (Lee et al., 2011).

**Table 2:** Updates on the development of GOD based enzymatic biofuel cell (EBCs).

**Description**	**References**
GOD from *P. funiculosum* 46.1 + HRP	Ramanavicius et al., 2015
rGOD from *P. amagasakiense* (wild type *Pa*GODwt + mutant *Pa*GOD (*Pa*GODmut) with poly(3,4-ethylenedioxythiophene)-graphene nanocomposite	Arribas et al., 2016
Entrapping cross-linked GOD aggregates within a graphitized mesoporous carbon	Garcia-Perez et al., 2016
Biofuel cell cathode with laccase-containing culture supernatant from *Pycnoporus sanguineus*	Fokina et al., 2015
Mediator-less glucose/oxygen based biofuel cell with laccase	Christwardana et al., 2016
Mediator-less DET type biofuel cell enabled with carbon nano-dots	Zhao et al., 2015
GOD + graphite particle with redox mediator compression	Zebda et al., 2012
GOD immobilized with polyaniline nanofiber	Kim et al., 2011
Immobilization of GOD on modified-carbon-paste-electrodes	Ambarsari et al., 2016
GOD immobilized through both cross-linking + physical entrapment	Chung et al., 2016
GOD conjugated with site-specific gold nanoparticle	Holland et al., 2011
GOD and bilirubin based electrodes	Kim et al., 2009
Cross-linked GOD clusters	Dudzik et al., 2013
Nano-tube ensemble films based GOD	Miyake et al., 2011
Covalent co-immobilization of GOD and ferrocene dicarboxylic acid	Shim et al., 2011
Co-immobilization of glucoamylase + GOD	Lang et al., 2014
Electrically wired polyphenol oxidase + GOD	Giroud et al., 2012
Graphene and multi-walled carbon nano-tubes (CNTs)	Devadas et al., 2012
GOD-CAT co-immobilized catalyst (CNTs/PEI/(GOD-CAT)	Christwardana et al., 2017
MET by biocatalytic anode of sulfonated graphene/ferritin/GOD layer-by-layer biocomposite films	Inamuddin et al., 2016

Another unique strategy used for stabilization of GOD is based on covalent glycosidation. Matos et al. (2012) chemically modified GOD by covalent glycosidation with cyclodextrin-branched carboxymethylcellulose (CMC-CD) polymers. The obtained neoglycoenzyme contained 0.78 mol of polysaccharide per mol of GOD and retained 67% of its initial activity. Further, it showed a better thermostability when compared with free enzymes, which increased from 45 to 51°C. In addition, derivatization of GOD with CMC-CD increased its resistance to inactivation at 45°C by 2.2-fold, protected the molecule against inactivation with the anionic surfactant sodium dodecyl sulfate to the point that it retained 75% of its activity after an incubation period of 3 h, and extended its pH tolerance toward alkaline pH (7.5). Therefore, this strategy was confirmed to be an effective strategy for enhancing the stability of this GOD.

### Mutagenesis and recombination

A number of efforts have been focused on the improvement of GOD production through the specific selection of fungal isolates using mutagenesis approaches and classical screening (Witteveen et al., 1990; Ramzan and Mehmood, 2009). Traditionally, strain development through mutagenesis requires a tedious and lengthy process as it requires sophisticated screening methods to identify and separate superior isolates among mutagen-treated populations. However, a significant advancement of screening methods is their simplicity because they do not require understanding the molecular and physiological aspects of manipulating the organism (Gromada and Fiedurek, 1997).

Mutagenesis of different fungi as a strategy for the improvement of GOD production has successfully improved enzyme activities by up to 77% (Ramzan and Mehmood, 2009). Further, expression and optimization of GOD production have been achieved through the application of recombinant DNA technology in fungi other than their native sources to overcome these difficulties. Cloning and overexpression of GOD in *Saccharomyces cerevisiae, Escherichia coli*, and other fungal hosts from *Aspergillus* and *Penicillium* species have been successfully carried out (Park et al., 2000; Kapat et al., 2001; Malherbe et al., 2003; Shaikh and Trivedi, 2016). Witt et al. (1998) cloned and expressed the gene encoding *P. amagasakiense* GOD in *E. coli*. Insoluble inclusion bodies were expressed as the activity of GOD following reconstitution, that resulted in the active enzyme to express the secondary structure composition and enzymatic properties similar to native *P. amagasakiense* (Witt et al., 1998). Park et al. (2000) expressed the *A. niger* GOD gene successfully in *S. cerevisiae* containing different promoters and terminators and showed that the hybrid yeast ADH2-GPD promoter had high amounts of GOD production. These possibilities dramatically increased the range of industrial applications for GOD with economic feasibility.

Several studies have revealed that GOD was one of the largest foreign proteins able to be expressed heterologously. Therefore, these expression systems are currently used for the expression of GOD. Although recombinant species of *Aspergillus* and *Trichoderma* and wild-type strains as hosts have shown significant results in the production of heterologous proteins (Nevalainen et al., 2005), since the morphology of mycelia represents dramatic engineering challenges during submerged fermentation (Wang et al., 2003). The presence of mycelia produces highly viscous broth during fermentation, thereby affecting the rate of agitation, pumping, and oxygen supply to the culture. In addition, the presence of mycelia also results in non-ideal mixing of the broth and poor nutrition to the fungi, resulting in the low yield recovery of the final product (Li et al., 2000). Owing to this, yeast species are considered preferable by the fermentation industries since they allow high biomass production and easy cell separation from the culture during submerged fermentation (Papp et al., 2006).

Various yeast species have been shown to be extremely useful for the expression and analysis of rigid, particularly *S. cerevisiae, Hansenula polymorpha*, and *Pichia pastoris*, which have been researched for the last several years for high-yield production of heterologous GOD with promising results. These organisms offer certain advantages over bacteria as a cloning host (Demain and Vaishnav, 2009). Specifically, they exhibit rapid media growth with high cell biomass, and have the ability to produce extracellular heterologous proteins with advanced genetics than other eukaryotes. *A. niger* GOD can be produced by *S. cerevisiae* at 9 g/L. Additionally, the *P. pastoris* yeast expression system was successfully used to produce active GOD originating from *A. niger* or from *P. variable* P16 that was not over glycosylated (Guo et al., 2010; Qiu et al., 2016). Naturally, *A. niger* or *Penicillium* spp. produce about 10% glycosylate as an extracellular enzyme called GOD (Ferri et al., 2011), whereas *S. cerevisiae* and *H. polymorpha* species of yeast produce recombinant enzymes with a concomitant reduction in enzymatic activities by hyperglycosylation (Romanos et al., 1992). *P. pastoris*, a methylotrophic yeast has been reported to be the most effective host for rGOD production (Zhou et al., 2001). These studies illustrated the high potential for enhanced production of GOD in yeasts.

*E. coli*-based production of non-glycosylated rGOD has been reported in an inactive apo-form (Witt et al., 1998), however, use of other cofactors for its active holo-form expression is still needed. Although reports have confirmed similar expression properties of recombinant enzymes to the native ones, their industrial applications still bear several limiting factors, such as their higher cost and deficient reconstitution. GOD secreted by yeast shows overexpression, which facilitates higher production and purification and helps in enzyme modification by random mutagenesis. A number of methods have been employed for the initial screening of GOD mutant lines using fungal cultures of the isolated clones (Zhu et al., 2006). Using an expression system of *S. cerevisiae*, GOD was found to show an improved catalytic reduction from *A. niger* (Zhu et al., 2007), which was also produced as a tagged and/or bi-functional fusion enzyme using the yeast expression system. However, limiting factors of using yeast include their less potency during large-scale GOD production due to α-1, 3-linked mannose residues and hyperglycosylation, resulting in the removal of strong and tightly-regulated promoters as well as causing an anti-genic response (Adrio and Demain, 2014).

### Enzymatic engineering techniques

Enzyme engineering using currently available methods such as rational design, rational redesign, and directed evolution can lessen or remove the limitations to the improvement of GOD variants. Rational redesign strategies were recently used to improve the catalytic function and stability of GOD. Holland et al. (2012) combined genetic elements from the two most widely studied GOD producers, *A. niger*, and *P. amagasakiense*, by rational re-design, to produce an enzyme possessing the strong catalytic capacity and stability. Fisher et al. (2014) also pointed out that by using a rational enzyme engineering approach, it is possible to construct a GOD having the stability of its homolog from *A. niger* and the catalytic activity of its homolog from *P. amagasakiense* to better carry out glucose oxidation in industrial applications. Recently, Song et al. (2016) improved the anti-oxidation properties of GOD against H_2_O_2_ (competitive inhibitor) by designing mutants in which leucine was replaced with methionine using computer-aided homology modeling with the CDOCKER algorithm. The results obtained were consistent with those of the computer-aided analysis, suggesting that this method may be useful for enzyme structure optimization.

The directed protein evolution has recently come into use in the molecular modification of GOD, and it is also expected to improve the yield of GOD. Zhu et al. (2006) employed, directed evolution to enhance the catalytic performance of GOD, which resulted in a 1.5-fold improvement in kcat. Ostafe et al. (2014) described an ultrahigh-throughput screening method for sorting out the best GOD variants generated by directed evolution that incorporated several methodological refinements such as flow cytometry, *in vitro* compartmentalization, yeast surface display, fluorescent labeling of the expressed enzyme, delivery of glucose substrate to the reaction mixture through the oil phase, and covalent labeling of the cells with fluorescein-tyramide. Similarly, Prodanovic et al. (2011) employed an ultrahigh-throughput screening system for GOD from *A. niger* by directed evolution in yeast cells. Recently, Horaguchi et al. (2012) utilized a rational amino acid substitution to engineer GOD with remarkably low oxidative activity and high dehydrogenase activity, which was higher than that of the wild-type enzyme. Marín-Navarro et al. (2015) proposed a combined method employing random and rational approaches to identify and structurally analyze amino acid substitutions that increased the stability and activity of *A. niger* GOD. Their results revealed structural motifs of the protein are critical to its stability.

Although fungal sources offer a wide range of enzymatic properties, new features such as marked product inhibition, higher product yields, or secretion signal could be designed into specific GOD using innovative tools of state-of-the-art protein engineering. The high-level progress of GOD production through the engineering of strains was recently reviewed by Liu and Piwu (2013). Similarly, Suraniti et al. (2013) successfully redesigned a key amino acid of GOD from *P. amagasakiense* using non-active site mutations to enable improved biotechnological applications, especially in EFCs. Optimization of GOD can be achieved by employing modern molecular level tools and techniques in association with bio-process engineering technologies that may result in economically feasible enzyme production system. Application of efficient recombinant microbial technologies on new resources of GOD and protein engineering technology have proved significant efficacy to convert GOD into a relevant synthetic tool. Thermostability of GOD may further enhance its industrial value in addition to its boosting functional food market.

## Applications of GOD in various sectors

### Applications in various food sectors

#### In baking industry

GOD is an efficient oxidant for the production of bread with improved quality and extended loaf volume in the baking industry (Rasiah et al., 2005; Wong et al., 2008; Steffolani et al., 2010). H_2_O_2_ produced by GOD yields more elastic and viscous dough (Vemulapalli et al., 1998). In addition, Vemulapalli and Hoseney (1998) reported the drying effects of GOD on the dough, which were mediated by the gel-forming ability of water-soluble pentosans, reduction in sulfhydryl content, and the increase of viscosity in the water-soluble dough. Also, GOD has been found to display protein cross-linking in the dough (Rasiah et al., 2005). GOD improves the quality of bread and strengthening of wheat dough when used as an additive (Bonet et al., 2006). GOD also enhances the viscoelasticity of dough (Kouassi-Koffi et al., 2014). Specifically, Kouassi-Koffi et al. (2016) assessed the effects of wheat dough viscoelasticity by adding GOD to predict final bread quality. However, enzymes must be added with care since undesirable effects can be caused by excessive enzymes. GOD, along with lipase also enhances the quality and shelf-life of pan bread (El-Rashidy et al., 2015). Dagdelen and Gocmen (2007) studied the effects of GOD along with ascorbic acid and hemicellulase on bread quality and dough rheology and found that bread quality is mainly dependent on original wheat flour quality, while dough rheology depended on the amount of enzyme. The combination of GOD, α-amylase, and xylanase on dough properties and bread quality were also studied by Steffolani et al. (2012). Kerman et al. (2014) investigated strengthening properties of wheat dough and quality enhancing parameters of wheat bread in response to the addition of GOD along with ascorbic acid. Similarly, da Silva et al. (2016) also verified the performance of xylanase and its interaction with GOD and ascorbic acid on the quality of whole wheat bread. Furthermore, the addition of basal additives as well as ascorbic acid (32%), α-amylase (4.2%), and GOD (63.8 %) to wheat flour, reduced crumb firmness and chewiness, as well as improved adhesion, elasticity, cohesion, and bread volume specifically (Kriaa et al., 2016). Recently, the synergistic effects of amyloglucosidase, GOD and hemicellulase utilization on the rheological behavior of dough and quality characteristics of bread was studied by Altınel and Ünal (2017). Decamps et al. (2013) revealed the molecular mechanism of dough and bread stability improvement by the pyranose oxidase from *Trametes multicolor* and GOD by *A. niger* during the cross-linking of gluten protein and arabinoxylan by the formation of H_2_O_2_. Aprodu and Banu (2015) studied the effects of *Psyllium*, pea fiber, oat bran, water, and GOD on rheology and baking properties of gluten-free bread made from maize, and it was suggested that GOD has significant ability to improve the specific bread volume for all types of fibers.

#### In beverage industry

GOD plays a novel role in the manufacturing of beverages because it is used to diminish the low alcohol substances of wine by eliminating the residual glucose that would otherwise be converted into alcohol through anaerobic fermentative processes. Moreover, the H_2_O_2_ produced during chemical processes imparts a bactericidal impact on acidic corrosive and lactic corrosive microbes amid the fermentative procedures. This process must be conducted by adding GOD prior to fermentation as GOD utilizes a portion of the glucose presents, making it inaccessible for liquor aging, bringing about wine with decreased alcohols and simultaneous generation of H_2_O_2_ that reduces the growth of fermentative microorganisms. The H_2_O_2_ produced could easily be removed from the system using CAT, which breaks it into oxygen and water. The bactericidal effect of H_2_O_2_ reduces the addition of other chemical preservatives in the wine (Malherbe et al., 2003). It has been well-demonstrated that pre-treatment of grape juice by GOD/CAT enzyme system can reduce alcohol fermentation potency significantly through conversion of available glucose to GA. Similarly, the technical feasibility of GOD/CAT enzyme system was investigated as an alternative to decrease the glucose concentration and eventually production of reduced red wine (Valencia et al., 2017). GOD has shown significant efficacy on determining glucose content in body fluid and effectively removes oxygen and residual glucose from beverages (Yildiz et al., 2005). Lopes et al. (2012) developed a biosensor composed of GOD and immobilized HRP, which efficiently determined glucose content in beverage samples such as orange juice, as well as energetic and sport drinks. GOD bound emulsified nano-particles of bovine serum albumin along with chymotrypsin results in soft drinks/non-alcoholic beverages being free from turbidity and opalescence while maintaining their pH as acid regulators (Sharma, 2012). Further, Mason et al. (2016) demonstrated the potential role of a novel glucose electrochemical biosensor based on the immobilization of GOD into a nylon nano-fibrous membrane (NFM) during analysis of glucose in commercial beverages and monitoring of the brewing process for making beer.

#### Use for production of dry egg powder

GOD has been used effectively to remove remaining glucose and oxygen from foods to extend their shelf-life (Zia et al., 2012). The reaction of protein amino group and reducing sugars is known as non-enzymatic Maillard browning, which results in the formation of unwanted flavor and undesirable browning in dried egg powder, suggesting prior removal of glucose content from the liquid egg before its drying (Sisak et al., 2006). Removal of glucose provides dried egg powder a prolonged shelf-life and increased microbial tolerance. Also, production of H_2_O_2_ by Maillard reaction helps to destroy unwanted microbes normally found in liquid egg (Dobbenie et al., 1995), while it can later be evacuated utilizing a moment catalyst, CAT, which changes over H_2_O_2_ to oxygen and water (El-Hariri et al., 2015). GOD/CAT is utilized to expel glucose amid egg-powder production for use in the baking industry, which prevents browning during dehydration brought about by the Maillard response, and to give slight improvements to the crumb properties of bread and croissants (Rasiah et al., 2005). Increasing browning mediated by the Maillard reaction is also a noteworthy aspect causing harmful effects to eggs and potato products. Application of GOD may provide sustainable results to reduce unwanted browning in the same manner (Low et al., 1989).

### Applications in various pharma/medicine sectors

#### Use of gluconic acid (GA) production in the food/pharma industry

One of the major applications of the GOD catalyzed reaction is the production of GA and its derivative salts. GA was found to play extensive roles in different sectors of food industries and utilized as a causticity controller, raising specialist, color stabilizer, an antioxidant and chelating operator in bread, feeds, beverages, and so on (Brookes et al., 2005). In dairy industries, GA is used for the cheese curd formation, improvement of heat stability of milk, prevention of milk stone, and cleaning of aluminum cans. However, GA is most widely used as acidulant, sequestrant as well as potential anti-oxidant in various industries (Golikova et al., 2017). In the pharmaceutical based industries, the metal derived Na, Ca, Zn, and Fe salts of GA is widely used in the synthesis of important drugs including sodium, calcium or ferrum gluconates, and glucono-delta-lactone which have diverse industrial applications (Ramachandran et al., 2006; Golikova et al., 2017). Sodium gluconate has great potential to chelate metal ions and can be used to remove bitterness from food stuff (Costa et al., 2015; Pal et al., 2016). Pharmaceutical application of calcium gluconate has been confirmed for the treatment of calcium associated deficiencies (Khurshid et al., 2013), whereas for the treatment of common cold, wound healing, and zinc-deficiency associated with delayed sexual maturation, infection susceptibility, mental lethargy, and skin rashes have been well-treated using zinc gluconate.

GA can be produced through biochemical, electrochemical, bioelectrochemical, and fermentative processes, although fermentative processes are preferred for GA production as other approaches are expensive and less productive (Wong et al., 2008; Pal et al., 2016). It has been reported that GA production through GOD catalyzed reaction is highly dependent on the substrates used, oxygen concentration and temperature (Ramachandran et al., 2006; Khurshid et al., 2013). Moreover, the catalytic efficiency for the conversion of glucose to GA is highly dependent on the stability of GOD. Recently, it has been demonstrated that enzymatic cross-linking with GLU modified on inorganic support (SiO_2_) system provides most active and stable system that causes the maximum (up to 85%) yield of GA (Golikova et al., 2017). Several studies have been conducted to optimize the production of GA. For example, Purane et al. (2011) optimized parameters such as glucose concentration, inoculum density and inoculum age for GA production using *P. chrysogenum* 724 and found that the maximum amount of GA produced (31.16 g/L) was reported at a glucose concentration of 100 g/L. Ping et al. (2016) demonstrated the effects of oxygen supply on intracellular flux distribution for enhanced production of sodium gluconate by *A. niger* and reported that the higher oxygen concentration was required for enhanced synthesis of GA. Further, Ramezani et al. (2013) reported the effects of hydrodynamic properties on kinetics parameters to achieve higher GA production by using GOD. It was found that the increased oxygen gas velocity resulted in the increasing rate of glucose oxidation reaction because of the higher transformation of oxygen from a gas to a liquid state. Optimization of mass transfer characteristics and operating condition during GA production with immobilized GOD, it was found that a bubble-column reactor had better mass transfer properties because it provided higher GA production under low GOD activity relative to other reactors. Recently, many investigations have investigated GA production using substrates through multi-enzymatic steps. Mafra et al. (2015) developed a method for GA production using sucrose as the substrate that was catalyzed by a multi-enzymatic system, including invertase, GOD, and CAT in an air-lift reactor. Similarly, Silva et al. (2011) reported the production of GA using sucrose and multi-enzymatic components in batch and membrane continuous reactors. GA production through fermentative processes also depends on morphological parameters of the fermenting organism used. Indeed, it was reported that the dispersed pattern of the mycelial morphology of *A. niger* resulted in increased GA production rather than pellet morphology (Lu et al., 2015). GA production through fermentative processes is the most widely accepted technique and the most common challenge to these methods is downstream processing (separation and purification). However, these hurdles can be resolved by membrane-based separation (Pal et al., 2016).

#### Use as an antioxidant/preservative agent

Many food products contain oxygen, which promotes bacterial growth. In canned/bottled/packaged food, it is important to maintain an anaerobic condition, thus, removal of oxygen is essential in packaged food products (Kirk et al., 2002). Karimi et al. (2012b) studied the removal of dissolved oxygen from water through the reduction of glucose, catalyzed by GOD and CAT enzymes. Moreover, GOD could be utilized for the removal of oxygen from the top of bottled beverages such as wine and beer to maintain the taste and flavor (Labuza and Breene, 1989; Wong et al., 2008).

Non-enzymatic browning of processed fruits and tomato puree can be controlled using the GOD/CAT system during storage. Food deterioration and rotting of high-fat nourishments, for example, mayonnaise and mixed greens dressing are associated with lipid peroxidation (Isaksen and Adler-Nissen, 1997), where the application of the GOD/CAT system has potential to retard lipid peroxidation during storage (Bankar et al., 2009b). The GOD/CAT system has the oxygen scavenging ability, thereby making oxygen unavailable for lipid metabolism during oxidation of glucose (Isaksen and Adler-Nissen, 1997). The overall GOD catalytic reaction consumes two glucose particles and an oxygen molecule, resulting in the production of two GA molecules. During the reaction, the consumption of oxygen allows GOD to be used as a strong antioxidant and scavenger of oxygen, thus facilitating its application as a food preservative due to stabilizing effect. Additionally, GOD has been effectively used in the food system as a strong stabilizer due to its oxygen removing ability and prevents color and flavor loss in a variety of beverages including canned fish, beer, soft and energetic drinks (Crueger and Crueger, 1990; Bhat et al., 2013). GOD can also be used instead of potassium bromate as an oxidizing agent in bread making (Moore and Chen, 2006).

#### Use in oral care

GOD and lactoperoxidase have been used in oral health-care products due to their antimicrobial potential (Afseth and Rolla, 1983; Güneri et al., 2011). The H_2_O_2_ produced by GOD functions as a valuable bacteriocide. *Streptococcus mutans*, which inhabits the oral cavity and causes tooth-decay, is carried by almost every human being. The ability of GOD to kill *S. mutans* can be improved by enzymatic fusion using heavy-chain antibodies (Etemadzadeh et al., 1985). Hill et al. (1997) reported that GOD and/or GOD with HRP encapsulated reactive liposomes were found to exhibit antibacterial effects against tooth decay bacteria in saliva, suggesting their potential efficacy in oral hygiene. Senol et al. (2005) reported the antibacterial activities of oral care products containing GOD against ventilator-associated pneumonia pathogens.

#### Use as an antimicrobial agent

GOD has antimicrobial activity against different foodborne pathogens. GOD has shown enormous potential to inhibit the growth of various foodborne pathogens, including *Clostridium perfringens, Campylobacter jejuni, Salmonella infantis, Staphylococcus aureus*, and *Listeria monocytogenes* (Tiina and Sandholm, 1989; Kapat et al., 1998; Cichello, 2015). Further, GOD covalently immobilized on biorientated polypropylene films was found to inhibit the growth of *E. coli* and *B. subtilis* (Vartiainen et al., 2005). Murray et al. (1997) reported inhibitory effects of culture filtrates of transformed *T. flavus* against *Verticillium dahliae in vitro* and found that the GOD secreted from *T. flavus* dramatically inhibited the microsclerotial and hyphal growth of the large proportions of *V. dahliae*. Malherbe et al. (2003) reported that the *S. cerevisiae* transformants harboring the GOD gene from *A. niger* exhibited antimicrobial efficacy in plate culture assay against lactic acid and acetic acid producing bacteria. Zia et al. (2013) also observed the antibacterial activity of GOD produced by *A. niger*. GOD produced by *P. chrysogenum* showed antifungal activity against different fungal pathogens (Leiter et al., 2004). GOD and its products such as H_2_O_2_ and GA showed *in vitro* antimicrobial activity against *Paenibacillus larvae* ATCC9545 (Sagona et al., 2015). Application of edible antimicrobial films has been approved to enhance the shelf-life of food products by releasing enough amount of antimicrobial substances on the surface of food products. The antimicrobial activity of the edible films containing antimicrobial agents, nisin (N), and/or GOD, into the matrix of whey protein isolate (WPI) films was assessed against *Brochothrix thermosphacta* (NCIB-10018), *Listeria innocua* (ATCC-33090), *E. coli* (JM-101), and *Enterococcus faecalis* (MXVK-22). The greatest antibacterial activity was observed in WPI films containing only GOD (Murillo-Martínez et al., 2013). The polyamide and ionomer films with immobilized GOD inhibited the growth of bacteria such as *E. coli* CNCTC 6859, *Pseudomonas fluorescens* CNCTC 5793, *Lactobacillus helveticus* CH-1, *Listeria ivanovii* CCM 5884 and *L. innocua* CCM 4030 on agar media (Hanušová et al., 2013). Recently, a new photo-dynamic glucose-based antimicrobial system encapsulating GOD, HRP, and BRET (bioluminescence resonance energy transfer) was developed for the inactivation of various bacterial and fungal pathogens through the network of organic and inorganic materials (Yuan et al., 2015).

#### Use as biosensors in medicine industry

Biosensors are widely used in the food industry, monitoring of environmental hazards, and clinical applications. GOD has been widely employed in glucose-based biosensors because of its high selectivity for glucose and functionality under extreme temperature, pH, and ionic resistance. Glucose biosensors for diabetic blood monitoring are very convenient, reliable, rapid, and accurate. Many studies have been conducted to develop sophisticated advanced technologies, and better alternatives such as point sample tests, and the continuous glucose monitor (CGM) are being developed (Wang and Lee, 2015; Sode et al., 2016). The CGM sensor has shown a significant role in diabetes as an alternative means while measuring blood glucose level and provides an alarming node on events associated with blood glucose metabolism (Wang and Lee, 2015). Further, in recent years, the drawbacks and limitations associated with glucose biosensors have been nullified using advanced approaches such as electrodes, membranes, enzyme immobilization, and nano-composite film modified electrodes.

The glucose biosensor system works on the concept of catalyzation of β-D-glucose oxidation by the immobilized GOD using molecular oxygen, resulting in the production of GA and H_2_O_2_. Amperometric glucose biosensors can be divided into three generations based on their operative principles. In the first generation biosensors, the concentration of oxygen/H_2_O_2_ is measured through an appropriate electrode and used as an indicator for glucose monitoring. The chemical reaction leading to oxidation of glucose causes depletion of oxygen or production of H_2_O_2_. The involvement of other redox species was the major problem associated with this generation. In second generation biosensors, mediators are involved in the backward and forward flux of electrons between the enzyme and electrodes, but this generation of biosensors had a low turnover rate and reduced proximity between the electrodes. Hence, these biosensors suffered from redox interferences (mostly oxygen). However, in the greatly advanced third generation biosensors, a method of direct electron transfer from enzyme to electrode was developed through “wired” relay centers (Wong et al., 2008) by immobilizing enzymes within the thin films with different modifications. The direct electron transfer process could be achieved through several modifications and the biosensors developed based on such modified electrodes have been found to have good reproducibility, selectivity, and stability for glucose oxidations. Velmurugan et al. (2015) reported the direct electron transfer reaction of GOD at gold nanoparticles-electro activated graphite/screen printed carbon electrode (AuNPs-EGr/SPCE).

The method of immobilization of GOD is considered a pivotal factor for the development of highly stable glucose biosensors with long-term operational life. The various methods by which GOD is incorporated into a biosensor include absorption, covalent attachment, cross-linking and micro-encapsulation (inert membrane entrapping enzyme into the transducer surface). Moreover, such immobilization could be achieved through one or in combination with others. Hong et al. (2016) developed biosensors based on GOD immobilized on graphene oxide (GRO) through various preparative approaches including enzyme adsorption (EA), enzyme adsorption and cross-linking (EAC), and enzyme adsorption, precipitation and cross-linking (EAPC). One of the most important aspects of such GOD based immobilization is the greater loading of enzymes for the efficient functioning which makes the overall system stable and selective in the form of cross-linking precipitated GOD aggregates. It has been reported that the problem of enzyme absorption on GOD based biosensors could be improved by using silicalite modified electrodes. The response of glutaraldehyde (GLU) cross-linked along with GOD adsorption on the silicalite-modified electrode (SME; GOD-SME-GLU) was found to be much more sensitive and reproducible than alone GOD-GLU based biosensors and have been employed in determining the concentration of glucose in juicy nectars and fruits (Dudchenko et al., 2016). Recently, the uses of carbon nano-chips (CNCs) have been used to modify the glassy carbon electrode for immobilizing GOD with the help of chitosan. These GODs/CNCs based system have increased the electrochemical response when used in GOD based bioelectronics devices (Kang et al., 2017). The properties of the immobilized enzyme in biosensors depend on both the enzyme and the supportive material involved (Ang et al., 2015). The two major limitations that restrict the immobilization of GOD on solid electrodes include inadequate electrical communications between the active sites of GOD and the surface of electrodes including enzyme leaching.

A huge range of electrode substrates has been used recently to overcome these problems which include metal-based nano-particles, carbon nano-tubes (CNTs), mesoporous silica, polymers, and sol-gels (Table 3). However, the electrocatalytic activities of GOD toward the oxidation of H_2_O_2_ in GOD based amperometric biosensors has been improved through the use of multilayered reduced graphene oxides (MRGO) sheets (Hossain and Park, 2017). In one such type of amperometric biosensor, GOD was immobilized onto MRGO sheets, and decorated with platinum and gold flower-like nanoparticles (PtAuNPs) modified Au substrate electrode. It has been found such fabricated MRGO/PtAuNPs modified hybrid electrode reveled higher electrolytic oxidation of H_2_O_2_ (Hossain and Park, 2017). Apart from these methods, the use of CNTs is considered a notable advancement in biosensing for the construction of glucose sensors due to their ability to promote the reactions of electron transfer of biologically significant biomolecules. Low-site-density based nano-electrodes aligning CNTs have been used for the detection of glucose (Lin et al., 2004), which showed good signal-to-noise ratio and detection limits.

**Table 3:** Recent developments in immobilization of GOD biosensor.

**Immobilization method**	**Description**
Biosensor based on GOD immobilized on different substrates/modified electrodes through physical absorption/chemical cross-linking/covalent attachment/microencapsulation	Chitosan submicron particles (Anusha et al., 2015); chitosan-based porous composite (Susanto et al., 2013); carbodiimide-treated activated carbon particles (Bailey and Cho, 1983); polyaniline film cross-linked with GLU (Gaikwad et al., 2006); polypyrrole-poly (vinyl sulphonate) composite film crosslinked with GLU (Çolak et al., 2012); nitrogen doped carbon dots electrodes (Ji et al., 2016); porous gold electrodes (Toit and Lorenzo, 2014); bulk and porous SiO_2_ (Libertino et al., 2008); poly (propylene imine) dendrimer (Shukla et al., 2013); screen-printed electrodes with organosilicon sol-gel matrix (Kamanin et al., 2014); microparticles based on poly-methacrylic acid (p-MAA; Pérez et al., 2016); aromatic redox probes intercalation (binding of redox-active tetraalkylammonium ions to DNA; Nguyen et al., 2016); GOD cross-linked with GLU adsorbed on silicalite modified electrode (GOD-SME-GLU; Dudchenko et al., 2016); GOD crosslinked with GLU and fluorescent oxygen films (Su et al., 2017).
Nanoparticles based nanocomposites for GOD immobilization	Graphite electrodes with colloidal gold nanoparticles (German et al., 2010); silicalite and nano beta zeolite (Soldatkin et al., 2015); co-immobilization with gold nanoparticles (Neto et al., 2015); nickel oxide nanoparticle (Salimi et al., 2007); nickel oxide nanoparticle modified carbon paste electrode (Erdem et al., 2013); Pt/functional graphene sheets/chitosan/silica nanoparticle (Wu et al., 2009); poly(methyl methacrylate)-bovine serum albumin core (PMMA-BSA)-shell nanoparticles (He et al., 2009, 2012); thiolated gold nanoparticles (Pandey et al., 2007); iron oxide magnetic nanoparticles (Abbasi et al., 2016); GOD + ETM immobilized on graphite pre-modified gold nanoparticles (AuNPs; German et al., 2015); GOD + fluorescent labeled gold nanoparticles (GOD-FLAuNPs; Muthurasu and Ganesh, 2016); carbon paste electrode (CPE) with zinc oxide (ZnO) nanoparticles (Shamsazar et al., 2016); gold nanoparticles-electroactivated graphite/screen printed carbon electrode (AuNPs-EGr/SPCE; Velmurugan et al., 2015); carbon coated nano tin sulfide assembled on glass carbon electrode (GCE; Chung et al., 2017).
Nanotubes /nanowires/nanofibers/nanorod-arrays	GOD-single wall carbon nanotube composites (Lyons and Keeley, 2008); double-stranded DNA single-walled carbon nano-tube hybrids (Xu et al., 2007); gold nanoparticles decorated graphene nanotubes (Devasenathipathy et al., 2015); graphene oxide-multiwalled carbon nanotubes hybrid (ERGO–MWCNT; Mani et al., 2013); single wall carbon nanotube (Tsai et al., 2009); multi-walled carbon nanotube (MWCNT)-titanate nanotube (TNT) nanocomposite (Liu et al., 2015); platinum modified carbon nanotube electrode (Tang et al., 2004); horseradish peroxidase + glucose oxidase cross-linked to multiwalled carbon nanotubes (Xu et al., 2014); ZnO nanowires + silicon nanowires (Miao et al., 2016); electrospun Mn_2_O_3_ nanofibers (Ding et al., 2011); nanorod arrays (ZnO) + gold nanoparticles (Zhao et al., 2015); GOD + carbon nano-chips (CNCs) + chitosan (Kang et al., 2017); ZnO nanorods based non-enzymatic fluorescent based biosensor (Mai et al., 2017); GOD immobilized on PVA/PAA nanofiber matrix (Kim and Kim, 2017).
Graphene-based GOD immobilization	Graphene oxide (Sehat et al., 2015); graphene-PPy (Alwarappan et al., 2010); metal decorated graphene (Baby et al., 2010); graphene/nafion (Chen et al., 2010); graphene-CdS (Wang et al., 2011); electrografting of thionine diazonium cations onto glassy carbon electrodes + graphene nanosheets (Shervedani et al., 2016); graphene quantum dots (Razmi and Mohammad-Rezaei, 2013).

The use of nano-materials in biosensors and bioelectronic devices has provided a new platform for efficient glucose monitoring due to their improved response time, high sensitivities, low detection limits, wide range linearity, and low power requirements. Recently, the real-time monitoring of glucose concentrations in GOD based biosensors has been achieved through the use of plasmonic nanoparticles/nanorods (Xu et al., 2017b). A list of major nano-materials, CNTs, and carbon nano-fibers (CNFs) used as electrical connectors between the electrode and the redox center, are given in Table 3. Wang X. et al. (2016) recently reported optimization and characterization of covalent immobilization of GOD using multi-walled carbon nano-tubes (MWCNTs) to maximize the loading of GOD, thereby, increasing the longevity of electric power or sensing signals.

#### Use for inducing resistance

GOD also plays an important role in the induction of defense responses in plants. Transgenic *Trichoderma atroviride* incorporating multiple copies of a GOD-encoding gene from *A. niger* was able to produce H_2_O_2_ after infection by fungal pathogens and showed higher antimicrobial activity against fungal pathogens as well as induced systemic resistance in plants (Brunner et al., 2005). Involvement of H_2_O_2_ during the plant resistance to bacterial disease agent was also revealed in *Arabidopsis* plant challenged by transconjugants of *Pseudomonas syringae* pv. *phaseolicola* expressing the avirulence genes *avrPpiA* and *avrPphB* matching the *RPM1* and *RPS5* resistance genes (Soylu et al., 2005). The application of genetic engineering tools has provided sustainable results in GOD expression in plants with increasing resistance to plants from bacterial infections (Wu et al., 1995). Further, Maruthasalam et al. (2010) reported that fungal GOD expression in transgenic tobacco plants provides them resistance from the cold by activating antioxidative defense system. Endogenous H_2_O_2_ levels of tobacco plants (*Nicotiana tobaccum* L. cv. SR1) were enhanced by constitutively expressing a GOD gene isolated from *A. niger*, and transgenic tobacco plants exhibited resistance to leaf spot fungal disease and bacterial wilt disease (Selvakumar et al., 2013). Similarly, the GOD gene from *A. niger* was inserted into potato plants, which resulted in leaves and tubers that produced high amounts of H_2_O_2_ constitutively and acquired resistance to the bacterium *Pectobacterium carotovorum* sub sp. *carotovorum* and the fungi *Phytophthora infestans* and *V. dahliae* (Bastas, 2014). Kachroo et al. (2003) also reported that GOD-overexpressing transgenic rice plants showed enhanced resistance to both *Magnaporthe grisea* and *Xanthomonas oryzae* pv. *oryzae*. Moreover, GOD isolated from *A. tubingensis* CTM 507 was found to have reduced spore formation, mycelial cord induction and mycelical vacuolization of pathogenic *Fusarium solani*. Hence, *A. tubingensis* CTM 507 suppressed the pathogenic attack and manifestation of disease in tomato (Kriaa et al., 2015).

### Applications in textile and energy production sectors

#### Use for enzymatic bleaching in the textile industry

Bleaching provides decolorization of natural pigments with a pure white appearance of the fibers. GOD has proven to be effective in the production of H_2_O_2_ for bleaching in the textile industry, the most effective bleaching agent of industrial significance (Bankar et al., 2009b; Saravanan et al., 2010; Mojsov, 2011; Soares et al., 2011). H_2_O_2_ produced by GOD decolorizes paprika dye effluent (Gonçalves et al., 2012) and malachite green (Karimi et al., 2012a). Moreover, the GOD application to the bleaching of textiles during upstream resizing and bio-scouring processes has shown promising results with the additional release of glucose (Buschle-Diller et al., 2002). The immobilization of GOD enzyme for the generation of H_2_O_2_ and its optimization has significantly affected the processing of bleaching in textiles. Tzanov et al. (2002) suggested the use of covalently-immobilized GOD on alumina and glass underpins for increased re-usage efficiency of enzymatic bleaching in textiles. The stability of GOD has been enhanced by a few immobilization procedures on different backings (Quinto et al., 1998; Tzanov et al., 2002; Blin et al., 2005; Betancor et al., 2006; Godjevergova et al., 2006). Recently, Aber et al. (2016) utilized a bio-fenton procedure for the decolorization of a dye-solution with *in situ* production of H_2_O_2_ by enzymatically catalyzed oxidation of glucose. They developed the optimal decolorization conditions by immobilizing GOD on magnetite nano-particles (Fe_3_O_4_) and reported that the best decolorization was achieved at a temperature of 10°C, pH of 6, GOD/support ratio of 1,800 U/g and time of 2.5 h. Under these conditions, they found that 450 U of GOD immobilized/grams of magnetite and that this system could be efficiently used for the oxidation of glucose and *in situ* generation of H_2_O_2_ for the removal of acid yellow 12. Farooq et al. (2013) compared conventional bleaching with GOD catalyzed bleaching of knitted cotton fabric and found that enzymatic bleaching led to better whiteness and mechanical properties such as tensile strength and tear strength. Furthermore, H_2_O_2_ produced by GOD was shown to be a significant alternative to the most extensively used commercial H_2_O_2_, in the textile processing industries. Moreover, Tzanov et al. (2001) found that the whiteness index of fabrics increased in response to a high concentration of glucose followed by a significant decrease in glucose concentration. However, the initial high concentration of glucose may cause discoloration of fabrics due to the presence of residual glucose. This problem can be overcome by using an excessive amount of GOD with an increased incubation time (Saravanan et al., 2010). Other important aspects of enzymatic processing in textiles are that the H_2_O_2_ generated during bleaching produces a comparable effect to scoured woven cotton fabric, while the GA produced acts as a chelator for metal ions, removing the need for use of an additional stabilizing agent (Tzanov et al., 2002). Additionally, the simultaneous application of GOD with peroxidases in the decoloration process improves bleaching of natural fibers (Opwis et al., 2008).

#### Use in improving biofuel production

**Figure 1:**
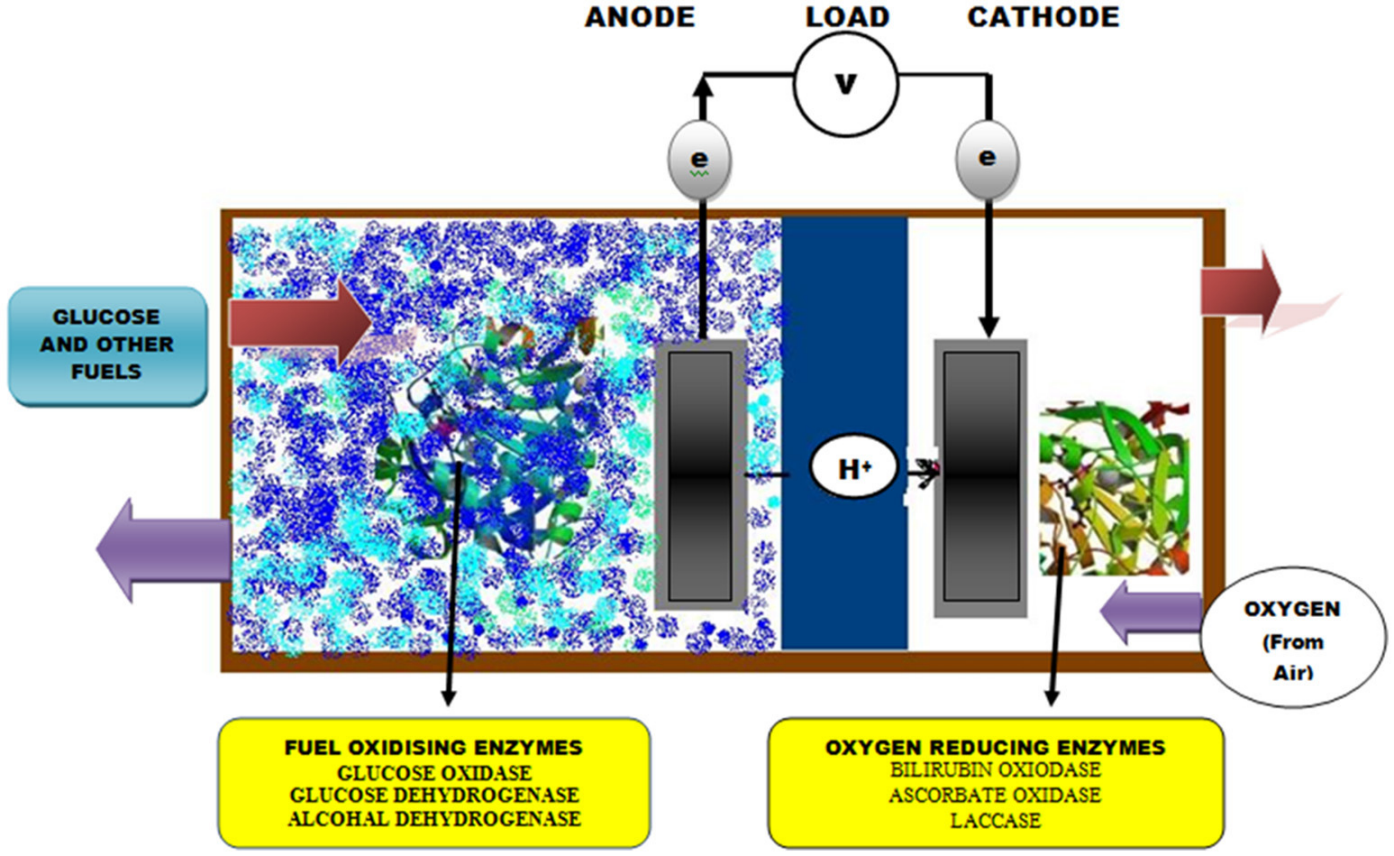
Generalized diagram of typical enzymatic biofuel cell (EBC) with associated components.

Biofuel cells (BFCs) use either enzymes or whole cell organisms as a biocatalyst to generate power directly from fuel substrates (glucose and ethanol). These enzyme-based systems are considered better alternatives for the development of future implantable devices (Sode et al., 2016). During the last decade, the rapid progress in enzyme-based BFCs allowed the development of membrane/compartment-less devices for miniaturization and use in implantable devices such as insulin pumps and glucose sensors in artificial pancreata and pacemakers (MacVittie et al., 2013; Falk et al., 2014). The design for manufacturing BFC is adjusted in such a way so that one electrode consisting of electro-conductive material is modified by a biocatalyst (enzymes) for specialized oxidation and reduction reactions. In one approach to the construction of BFCs, the catalytic reactions occurring at the anode are either mediated by GOD or glucose dehydrogenase and coupled with reduction reactions at the cathode mediated by di-oxygen reducing enzymes such as laccases, bilirubin oxidase or cytochrome oxidase (Barrière et al., 2006; Figure 1). More recently, efforts have been put forward to improve the catalytic efficiency of this system several folds by co-immobilization of GOD with other enzymes such as CAT. Christwardana et al. (2017) developed membrane less glucose biofuel cells (GBFCs) system for enhancing the power generation of membrane-less BFCs using GOD-CAT co-immobilized catalyst (CNT/PEI/(GOD-CAT) and reported that the system have increased the biocatalytic efficiency of GBFCs due to some synergistic mechanisms including removal of harmful H_2_O_2_ moiety by CAT and the simultaneous activation of GOD based desirable reactions.

Most of the output voltage and current signals in a typical BFC depends on the concentration of fuel, hence, enzyme-based BFCs could serve as an alternative means of an enzyme sensor system (Katz et al., 2001). Recently, there has been a great deal of efforts toward the development of bio-electrochemical devices based on unique enzymes. The presence of electron transfer sub-units or domain in these enzymes imparts specificity to directly transfer electrons to the electrodes during a bio-catalytic reaction (Tsugawa et al., 2012).

**Figure 2:**
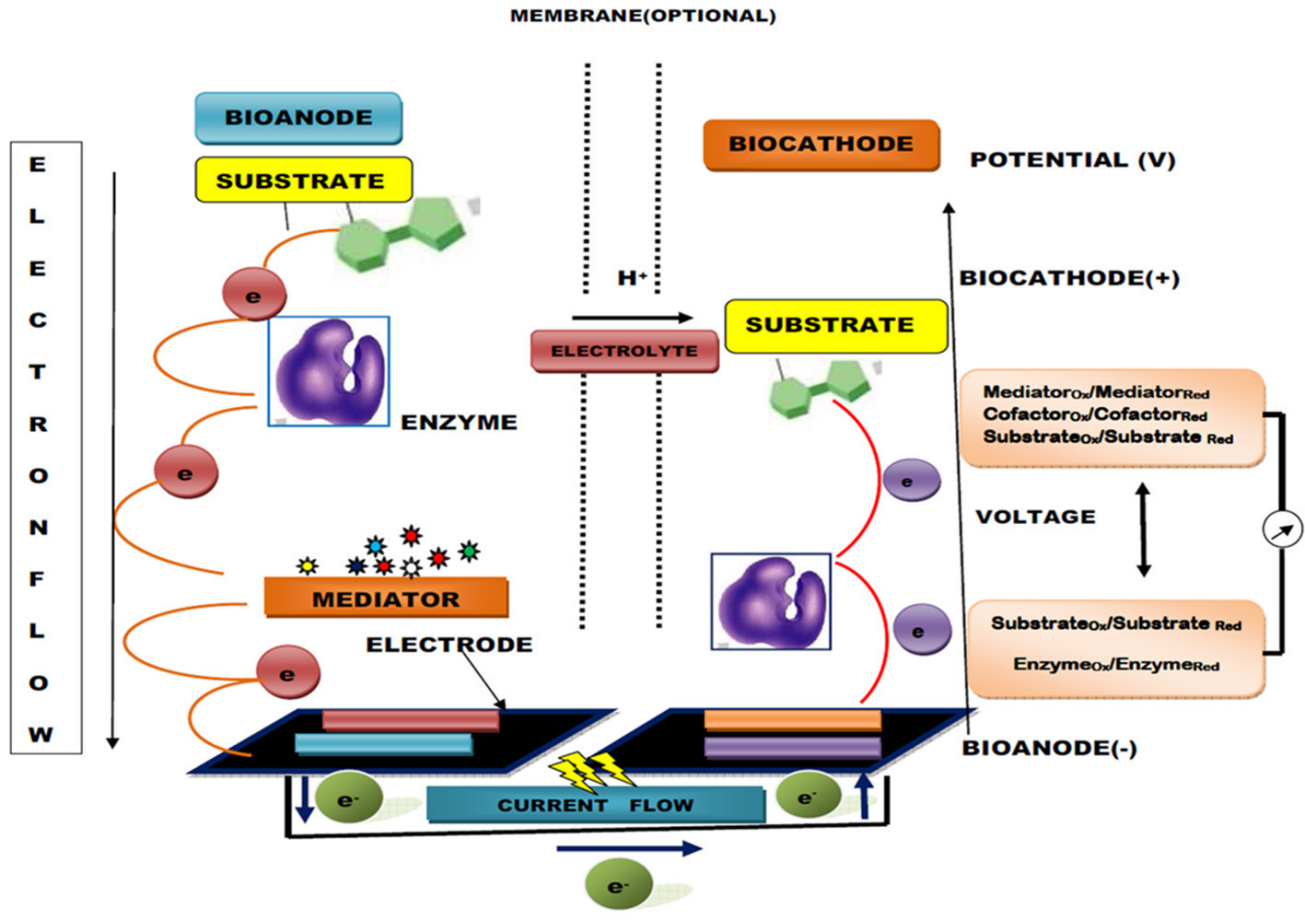
Generalized diagram of an enzymatic biofuel cell (EBC) with a mediator bioanode and direct electron transfer (DET) based biocathode (mediators are involved in fluxing electron flow between enzyme and electrode).

The sensitivity and conversion efficiency of BFCs are highly determined by efficient electron transfer occurring at the enzyme active center and electrode interface. However, these critical factors of sensitivity and conversion efficiency lead to difficulty in GOD catalysis based BFCs because the redox center inside this enzyme is buried inside the structure, a long way from any feasible electrode binding site (Sode et al., 2016). This problem can be resolved by using artificial electron acceptors and mediators or by precisely modifying the electrode surface with nano-scale conductive materials. The mediator assists in this electron transfer reaction through a mediator electron transfer (MET) mechanism by using little redox dynamic particles/polymers as electron bearers (arbiters) to/from one electrode to another or bio-catalytic site (Barton et al., 2004; Figure 2). These mediators can be polymerized specifically onto the surface of electrodes or co-immobilized with GOD to facilitate the rate of electron transfer by several-fold. Recently, a novel method entrapping cross-linked aggregates of GOD within a graphitized mesoporous carbon (GMC) network has been reported for the production of GOD nano-composites with an ability to provide the maximum rate of electron transfer and high electrical conductivity (Garcia-Perez et al., 2016). In contrast, the application of CNT immobilized GOD has given promising results (Ivnitski et al., 2007). Nano-carbon functionalization has shown perfect compatibility with other biological and chemical approaches with enhanced enzymatic functionality in implanted BFCs. Babadi et al. (2016) found that they could generate biocatalyst either by direct transfer of electrons or redox mediators for electron transfer. Some recent approaches to the development of enzyme-based BFCs are listed in Table 2.

The use of immobilization facilitates retention of biomass in reactor geometry, enabling their economic reuse and development of the continuous process. The technique also improves stability and prevents product contamination, paving the way for use of crude enzyme preparations such as whole cells in the bioprocessing (D'Souza, 2002). However, limitations such as lower power supply and reduced theoretical voltage (limited by the redox potential of cofactors and/or mediators employed in the anode and cathode) of a single BFC make it inadequate for providing power to any biomedical devices. Therefore, bio-capacitors have been developed using charge pumps connected to fuel cells. This novel approach has generated high voltage with sufficient temporary currents to operate an electric device without changing the design and construction of the EFC (Sode et al., 2016). The limited life expectancy of BFCs could be enhanced using technologies that favor enzyme stability, whereas power supply could be resolved by improving catalytic efficiency using directed evolution or other protein engineering methods.

### Other miscellaneous applications

In addition to the above-mentioned benefits, GOD could be used in some miscellaneous applications. For example, GOD has been used in several immunoassays and staining techniques and shown to have an ability on the removal of an excess level of glucose (Megazyme, 2003). From the geochemical prospects, GOD can be used for the preparation of leaching solutions, since both H_2_O_2_ and GA produced by GOD have a significant role in leaching (Wong et al., 2008). In medical sectors, GOD is widely employed in the monitoring of diabetic patients to measure their blood glucose activities using finger-prick blood samples. In addition, GOD induces several apoptosis characteristics viz, mitochondrial dysfunction, accumulation of Bax and release of cytochrome C in mitochondria, accompanied by activation of caspase-9 and caspase-3 (Rost et al., 2007; Kumar and Sitasawad, 2009; Yu et al., 2016). Moreover, GOD was also reported to induce cellular senescence in immortal renal cells through integrin-linked kinase (ILK) by downregulating *Klotho* gene expression, an aging- related kidney-secreted hormone with antioxidant properties (Yamamoto et al., 2005; Troyano-Suárez et al., 2015). Apart from these applications, it has been reported that the enzymatic activity of the white rot fungi *P. chrysosporium* is very effective in the presence of GOD and could be used in the biodegradation of lignin, recalcitrant pollutants, pulping and bleaching treatments (Ansari et al., 2016). Wang C. et al. (2016) developed a chemiluminescence (CL) amplification platform based on hollow structural calcium carbonate (HCC)/lucigenin and GOD (HLG) film and found that the GOD immobilized in the confined space of HCC particles exhibited improved biocatalysis. The visual CL bio-platform showed outstanding performance with high selectivity, a wide linear range and a low detection limit for sensing trace glucose. Recently, Nascimento et al. (2016) suggested the use of single cell “glucose nano-sensors” as nano-pipettes for tracing out cancer cells from normal cells. These nano-pipettes functioned as specifically developed nano-sensors to measure the glucose level in single cells with temporal and spatial resolutions. The covalently immobilized GOD at the sensor tip interacted with glucose forming GA, which was measured as the change in impedance due to the drop in the pH. Bandodkar et al. (2016) suggested the use of wearable chemical sensors and showed their promising effects in continuous monitoring of the user's health and fitness.

Diets supplemented with GOD have been reported to enhance growth performance, increase the contents of growth and development-related hormones and improve the fecal microflora of growing piglets (Tang et al., 2016). These changes might be attributed to the functional activities of GOD in the gastrointestinal tract, which utilizes O_2_ and produces H_2_O_2_ and GA. Using a similar approach, Tang et al. (2013) found that the contents of serum related hormones, intestinal health and growth performance of piglets can be effectively improved by a diet supplemented with 100 g/t GOD. However, more research is needed to determine the various effects of dietary GOD supplementation on other parameters of intestinal health. Moreover, during localization microscopic studies, GOD was used in single molecule localization microscopy (SMLM) buffers to decrease the solution oxygenation as well as to prevent fluorophore photobleaching (Szczurek et al., 2017).

## Future demands and implementations

The above review explains the potential applications of GOD in various industries with increasing demand in the food and flavoring, pharmaceutical, biotechnology and bioelectronic sectors. With a predicted annual growth rate of 7.6% per annum, the market value of the GOD enzyme increased to $6 billion by 2011 (The Freedonia Group, 2007). Although a plethora of microbial resources are currently available for efficient production of this enzyme, only a small fraction of microbial entities, particularly some selected strains of fungi and yeast, is currently exploited for the production of the enzyme. Further, huge attention should be paid on finding new sources of GOD and to develop cost effective fermentative processes for the efficient production and commercial exploitation of the enzyme. However, we currently do not have sufficient information available regarding the commercial production of GOD through fermentative processes and its further subsequent uses in different industries. Recently, enzyme immobilization processes and other technological innovative approaches have drawn scientific attention because of the potential for developing novel methods for the GOD production at large scale. However, problems such as diffusional constraints and decreased enzyme activity after immobilization have further limited the uses of immobilized GOD and therefore need to be improved to achieve greater benefits. The recent advancements in the field of bioinformatics can also revolutionized the field of GOD immobilization through docking and molecular dynamics techniques which can provide detailed information about the enzyme and ligands interaction. Further, the advances in DNA and RNA sequencing and their bioinformatic analysis can also provide novel insight into the structure and function of GOD. Large scale and efficient uses of GOD in various industrial sectors could be achieved through the use of modern biotechnological approaches such as concoction adjustment of existing chemicals through protein designing, site-directed mutagenesis, and recombinant expression of GOD genes in other potential microbes that could be further used at large scale to meet future demands. Fortunately, the different recent trends as mentioned throughout this review suggest that we are on the path of establishing a worldwide bio-based economy, and GOD may have a great contribution in this context.

## Author contributions

MD provided the general concept, and drafted part of the manuscript. MD, AZ, MA, and MM wrote part of the manuscript. MA, MD, LA, and SiS provided the necessary figures and tables. ShS, RU, RM, and VB also helped in the preparation of the manuscript and provided the necessary supervision. All authors revised and approved it for publication.

### Conflict of interest statement

The authors declare that the research was conducted in the absence of any commercial or financial relationships that could be construed as a potential conflict of interest.

## Abbreviations

GOD: Glucose oxidase
GA: Gluconic acid
FAD: Flavin adenine dinucleotide
H_2_O_2_: Hydrogen peroxide
rGOD: Recombinant glucose oxidase
CAT: Catalase
SSF: Solid-state fermentation
SmF: Submerged fermentation
EFCs: Enzyme fuel cells
BFCs: Biofuel cells
NFM: Nano-fibrous membrane
BRET: Bioluminescence resonance energy transfer
CGM: Continuous glucose monitor
CNTs: Carbon nano-tubes
CNCs: Carbon nano-chips
MET: Mediator electron transfer
GMC: Graphitized mesoporous carbon
CL: Chemiluminescence
GLU: Glutaraldehyde
HRP: Horseradish peroxidise.
